# Functional outcomes and subjective recovery of jumping to conclusions in schizophrenia

**DOI:** 10.1016/j.scog.2021.100212

**Published:** 2021-08-02

**Authors:** Seiichi Watanabe, Takamichi Taniguchi, Motoko Sugihara

**Affiliations:** a Department of Occupational Therapy, Medical Corporation Nasukougen Hospital, 375 Takakukou Nasu-machi, Tochigi, 325-0001, Japan; b Division of Occupational Therapy, Doctoral Program in Health Sciences, Graduate School of Health and Welfare Sciences, International University of Health and Welfare Graduate School, Tochigi, 324-0011, Japan; c Graduate School of Health and Welfare Sciences, International University of Health and Welfare, 2600-1 Kitakanemaru Ohtawara, Tochigi 324-0011, Japan; d Graduate School of Health and Welfare Sciences, International University of Health and Welfare, 4-1-26 Minato-ku Akasaka, Tokyo 107-8402, Japan

**Keywords:** Jumping to conclusions, Quality of life, Personal recovery, Schizophrenia, Japan

## Abstract

This study investigated the effects of the bias known as jumping to conclusions (JTC) on objective functional outcomes as well as subjective assessments of quality of life (QoL) and personal recovery among a sample of patients diagnosed with schizophrenia. Specifically, this study assessed the variables of JTC, psychiatric symptoms, neurocognitive functioning, objective interpersonal and daily activities, vocational domains, subjective QoL, and personal recovery among 94 participants. Results showed that those in the JTC group had significantly lower neurocognitive and functional outcomes (moderate effect sizes); however, subjective measures such as QoL and personal recovery did not differ significantly according to JTC (small effect sizes). After adjusting for attributes, there were no statistically significant differences, but the JTC group demonstrated lower overall functional outcomes and higher individual recovery, each with a moderate effect size. This ‘trade-off’ is not evidence-guaranteed, and further research is recommended to examine the relationship between social functioning and personal recovery in people with JTC bias.

## Highlights

• Those with jumping to conclusions (JTC) have lower overall functional outcome.
• JTC may be related to the functions of interpersonal relationships and daily life.
• When attributes were controlled, those with JTC had higher subjective recovery.
• The JTC group showed a gap between low functional outcome and high recovery.
• The above gap suggests a trade-off relationship between factors.

## 1 Introduction

The cognitive bias known as jumping to conclusions (JTC) is found in 40–60% of patients with schizophrenia (Dudley et al., 2016; Garety et al., 2013; Moritz and Woodward, 2005). Specifically, JTC can be defined as the tendency to make judgements based on small amounts of information (Dudley et al., 2016; Garety and Freeman, 1999). Comparing patients with psychosis to healthy participants, the difference in the amount of information collected is considered to be moderate (Hedges' g = 0.6) (So et al., 2016). While the general consensus is that JTC contributes to delusion formation and development (Garety et al., 2013), improvements in JTC are not necessarily accompanied by the mitigation of delusion (Garety et al., 2015). The results of a prior meta-analysis suggested a weak correlation between information gathering and delusion in the context of probabilistic reasoning tasks, and argued that JTC is not a necessary condition for delusion (Dudley et al., 2016). Previously, Garety and Freeman (1999) stated that JTC is ‘a property that represents a debt to delusion’ (p. 329), indicating that the relationship between delusion and JTC may be partial. Regarding neural mechanisms of JTC, evidence suggests that the prefrontal cortex may be implicated (Lunt et al., 2012). Specific neurocognitive effects have also been shown on verbal and working memory (Garety et al., 2013; Klein and Pinkham, 2018; Krezolek et al., 2019; Ochoa et al., 2014; Takeda et al., 2018). However, a decrease in JTC is not necessarily accompanied by an improvement in neurocognitive function (Moritz et al., 2015). Decreasing JTC may include changes in functional outcomes, especially vocational functioning and subjective quality of life (QoL) (Andreou et al., 2014; Moritz et al., 2011). Thus, it is unclear whether JTC is a ‘trait’ of schizophrenia or a consequence of neurocognitive dysfunction; regardless, making hasty decisions or conclusions without sufficient evidence or ignoring important evidence can cause problems across social and work settings and affect the individual's subjective assessments, such as QoL (Moore and Sellen, 2006).

Previous studies have examined how JTC impacts real-life concerns, including functional outcomes and subjective QoL. For example, it has been reported that the prognosis of functional achievement or functional outcome at 2–4 years is worse in patients with JTC at first onset of psychosis and when at clinical high-risk for psychosis (Catalan et al., 2020; Rodriguez et al., 2019). Furthermore, Andreou et al. (2014) assessed neurocognitive functioning, functional achievement, and QoL in patients affected by JTC, and found that improvements in JTC were significantly associated with improved employment status. This suggests that JTC improvements result in better functional outcomes, which is relevant to real-life and daily functioning. Additionally, JTC is a target for rehabilitation, making it important to understand its association with functional outcomes and subjective ratings (Moritz and Woodward, 2007; Penn et al., 2007).

A previous study (Andreou et al., 2014) examined the relationship between JTC and both functional achievement and subjective evaluations. However, no prior study has evaluated the relationship between JTCs and distinct domains of functional outcomes, such as interpersonal relationships, daily life functioning, and vocational functioning (Harvey, 2013). Therefore, this study aimed to determine whether there is a difference in functional outcomes and subjective evaluations between patients with schizophrenia with and without JTCs. This study assessed functional outcomes including neurocognitive functioning, interpersonal relationships, daily living, and employment, as well as subjective QoL and subjective personal recovery, in patients with and without JTCs, in a cross-sectional manner. It was hypothesised that functional outcomes and subjective ratings would be compromised by the tendency of the JTC group to collect less information and make more errors than the non-JTC group (Andreou et al., 2014; Moore and Sellen, 2006).

## 2 Methods

### 2.1 Participants

In total, 96 outpatients with schizophrenia, according to ICD-10 diagnostic codes, were recruited from the Nasukougen Hospital outpatient clinic. Data were collected from December 2019 to November 2020. The required sample size, which was 96 patients, was calculated using G*Power 3 (Faul et al., 2007), assuming an effect size (ES) of 0.58 (Dudley et al., 2016), α of 5% and power of 80%. Sampling was non-probabilistic, based on a list of patients. In total, 263 patients were screened by the psychiatrist they were consulting for acute conditions, strong depressive symptoms, and anxiety, and by considering the potential impact on the patient of their participation in the study; patients with strong psychiatric or mood symptoms were excluded. The inclusion criteria were: at least 20 years of age and able to understand and consent to participation in this study. The exclusion criteria were: current substance dependence, dementia, visual impairments, severe brain damage (e.g. from stroke), head injuries, and pregnancy. Of the remaining 171 patients, 96 agreed to participate in the study, and data from 94 patients were included in the analysis, excluding those who did not complete the bead task (Table 1).

**Table 1:** Demographic data for the sample population (*n* = 94).

	Mean	%	SD
Age (years)	48.4		12.8
Gender (m (%) f)	49	(47.9)	45
Education (years)	11.7		2.0
Duration of illness (years)	15.6		11.3
Antipsychotic medication dose (mg)	471.3		327.4
Housing situation (%)	38 (41.7)		
Protected work (%)	19 (20.2)		
Employed (%)	25 (26.6)		

This study was approved by the Institutional Review Board of the International University of Health and Welfare. Participants received written and verbal explanations of the study procedures and provided written informed consent.

### 2.2 Procedure

After providing consent, participants received a series of clinical assessments. Psychiatric symptoms were assessed by the psychiatrist in charge of the patient, while neurocognitive functioning was assessed by the author using the Trail Making Test, Japanese edition (TMT-J) (Ishiai, 2019). Functional outcomes were assessed by the author, the in-charge nurse, and health care providers, and confirmed by co-residents and others when necessary. Housing status was assessed as living alone and living with family while employment and school status were assessed as protective employment or employment. Protective employment was defined as employment transition support, continuous employment support, and employment for persons with disabilities included in the benefits of relevant welfare services. Employment was defined as part-time or full-time.

### 2.3 Assessments

Demographic data and disease information were obtained by perusing the medical records of participants. Participants also completed the following measurement scales.

#### 2.3.1 The brief positive and negative syndrome scale (Brief PANSS)

The Brief PANSS was developed by Yamamoto et al. (2010) and consists of six of the 30 items from the original PANSS (Kay et al., 1987): delusion, suspiciousness, emotional-withdrawal, passive/apathetic-social-withdrawal, tension, and unusual-thought-content. Yamamoto et al. (2010) also confirmed high concurrent validity between the Brief PANSS and original PANSS.

#### 2.3.2 JTC

Based on the experimental procedures conducted by Huq et al. (1988), this study evaluated JTC through a probabilistic reasoning assessment known as the Bead Task. Participants were presented with two jars, each containing beads of two different colours but mixed in opposite ratios. In this study, two tasks with two different ratios were used, with ratios of 85:15 and 60:40 (Jolley et al., 2014). For example, in the 85:15 task, one container with 85 yellow/15 black beads and one container with 15 black/85 yellow beads were used. Both containers were then hidden, and participants were informed that the beads would be individually removed from one of the containers. The beads were removed in a predetermined order (Huq et al., 1988; Jolley et al., 2014); each time one was drawn, the participant was asked to decide which container was being used or request that another bead be drawn. In this study, the focus was on the amount of information collected and evaluated in relation to JTC using the draws-to-decision (DTD) method (Fine et al., 2007). The Bead Task was created and reproduced in Microsoft PowerPoint and projected on a computer screen. Here, the maximum number of draws was 20, with the definition of JTC set as follows; non-JTC = DTD ≥ 3, JTC = DTD ≤ 2, (Dudley et al., 2016). In contrast, the 60:40 task was directly used to evaluate the presence/absence of JTC, as previous research has suggested its efficacy in this regard (So et al., 2012).

#### 2.3.3 TMT-J

The TMT-J is a standardised cognitive function assessment with age-specific data, designed for use in Japan (Ishiai, 2019). The TMT-J contains a Part A (TMT-A) and Part B (TMT—B), as in conventional TMT. In this study, the TMT-A assessed the processing speed (Laere et al., 2018), whereas the TMT-B assessed working memory (Pukrop et al., 2003; Ishiai, 2019). The shorter the time required, the higher the corresponding cognitive function. The validity of the TMT-A and TMT-B showed a significant correlation of 0.5–0.6, which was observed with the composite score of The Brief Assessment of Cognition in Schizophrenia (BACS; Mazhari et al., 2014).

#### 2.3.4 The specific levels of functioning scale - Japanese version (SLOF-J)

The SLOF is a functional outcome assessment scale developed by Schneider and Struening (1983). The SLOF-J was later developed by Sumiyoshi and Sumiyoshi (2012). It consists of 24 items across three domains: interpersonal functioning, everyday activities, and vocational functioning. Each question is rated on a scale ranging from 1 to 5 in terms of frequency of action and level of independence. Here, higher total scores indicate better functional outcomes. The criterion-related validity of the SLOF-J was confirmed by Sumiyoshi et al. (2016).

#### 2.3.5 The Japanese version of the 24-item recovery assessment scale (RAS-J)

The RAS-J is the Japanese version of the 24-item scale created by Chiba et al. (2010). Responses are rated on a five-point Likert scale ranging from 1 (strongly disagree) to 5 (strongly agree). For example, respondents are presented with prompts such as ‘I have a desire to succeed’. Higher total scores indicate greater recovery. The RAS-J was confirmed as both valid and reliable by Chiba et al. (2010). Cronbach's alpha for the scale in this study was 0.90.

#### 2.3.6 The Japanese schizophrenia quality of life scale (JSQLS)

The SQLS is a subjective, disease-specific, self-administered health-related QoL rating scale developed by Wilkinson et al. (2000). The SQLS was later translated into Japanese (JSQLS) by Kaneda et al. (2002). It assesses three domains: psychosocial relationships, motivation/vitality, and symptoms/side effects. The scale consists of 30 items (e.g. ‘Feel hopeless’) that respondents rated on a scale ranging from 0 to 4, with lower scores reflecting greater QoL. The reliability and validity of the JSQLS has been examined by Kaneda et al. (2002). Cronbach's alpha in this study was 0.97, 0.83, and 0.90 for the aforementioned three domains, respectively.

### 2.4 Statistical analysis

Participants were divided into non-JTC (DTD ≥ 3) and JTC (DTD ≤ 2) groups. Unpaired *t*-tests were used to compare group averages for continuous variables, while chi-square tests were used to compare group proportions for categorical variables. For the ES, Hedges' g and φ (Fritz et al., 2012) were calculated. Little's (1988) missing completely at random (MCAR) test was also performed, with missing values allowed at <5%. Based on the univariate analysis results, the significantly different variables (*p* < .05) were adjusted by propensity score. After propensity score matching, the same procedure was used for the analysis. In the analysis, the calliper was defined as the standard deviation of the logit-transformed value of the propensity score estimate multiplied by 0.2 (Austin, 2011). The ES was classified according to the general definition (Hedges' g: small = 0.2, medium = 0.5, and large = 0.8; φ: small = 0.1, medium = 0.3, and large = 0.5; Becker, 2000). All statistical analyses were conducted using IBM SPSS version 27.

## 3 Results

The participants were divided according to the presence or absence of JTC (non-JTC group: DTD ≥ 3, *n* = 40; JTC group: DTD ≤ 2, *n* = 54) and 57.4% exhibited JTC. No variable had a missing proportion greater than 5%, and Little's MCAR test determined that missingness was completely random (*χ*^*2*^ = 0.0, *p* = 1.0). Approximately 42% of the participants lived alone or with a partner (χ2(1) = 0.46, *p* = .83). Approximately 20% had protective employment and 27% had general employment, which were not associated with the presence of JTC (χ^2^(1) = 0.32, *p* = .61 and χ^2^(1) = 0.41, *p* = .64, respectively). Table 2 shows the statistical values of the sample groups and the results of the difference tests and ES. The JTC group had significantly lower scores for TMT performance and all functional outcome domains than the non-JTC group, with moderate ES. In contrast, there were no significant differences according to the RAS-J and JSQLS (small ES). However, this result also included the possibility that age, years of education, and duration of illness were confounding factors for participants in the JTC group. Table 3 shows the results after adjusting these variables via propensity score matching. The only variable with a statistical difference was DTD; variables with small to medium ESs included the SLOF-J total, RAS-J, and JSQLS symptoms/side effects.

**Table 2:** Comparison of participants' demographic characteristics in non-JTC and JTC groups.

	non-JTC (*n* = 40)		JTC (*n* = 54)		*χ*^2^ value/ *t*-value	*df*	*p*-value	ES	ES 95%CI
	Mean/%，SD		Mean/%，SD						
Age (years)	43.0	11.1	52.5	12.5	3.83	92	.000	0.81	0.37 to 1.22
Gender (m(%)f)	20 (50.0) 20		25 (46.3) 29		0.13	1	.835	0.04*	
Years of education	12.3	1.7	11.4	2.1	-2.19	90.5	.032	-0.45	-0.87 to -0.04
Duration of illness (years)	10.3	6.8	19.6	12.4	4.66	85.5	.000	0.89	0.47 to 1.31
Antipsychotic medication dose (mg)	416.2	314.2	512.1	333.8	1.41	92	.161	0.29	-0.12 to 0.70
Brief PANSS total	16.5	7.2	16.6	6.1	0.09	92	.928	0.02	-0.39 to 0.42
Brief PANSS positive	4.5	2.8	4.5	2.6	0.06	92	.955	0.01	-0.39 to 0.42
Brief PANSS negative	6.5	2.5	6.5	2.5	-0.22	92	.825	-0.05	-0.45 to 0.36
Brief PANSS general	5.4	2.7	5.6	2.2	0.42	92	.679	0.09	-0.32 to 0.49
DTD (85:15)	6.4	6.1	1.5	0.8	-4.98	40.1	.000	-1.19	-1.63 to -0.75
DTD (60:40)	8.1	6.3	1.4	0.6	-6.73	39.5	.000	-1.62	-2.10 to -1.15
TMT-A	51.2	23.1	66.0	40.5	2.06	91	.042	0.43	0.02 to 0.84
TMT-B	87.5	38.7	112.0	61.8	2.33	90	.032	0.46	0.04 to 0.87
SLOF-J total	99.4	12.8	91.1	13.9	-2.92	92	.004	-0.60	-1.02 to -0.19
SLOF-J Interpersonal Functioning	26.8	4.9	24.5	5.6	-2.07	92	.042	-0.43	-0.84 to -0.02
SLOF-J Everyday Activities	49.7	5.4	47.1	6.0	-2.18	92	.032	-0.45	-0.86 to -0.04
SLOF-J Vocational Functioning	23.9	4.5	21.5	5.1	-2.31	92	.023	-0.48	-0.95 to -0.01
Housing situation, (%)	25 (63.0)		30 (55.6)		0.46	1	.532	-0.07*	
Protected work, (%)	7 (17.5)		12 (22.2)		0.32	1	.614	0.06*	
Employed, (%)	12 (30.0)		13 (24.1)		0.41	1	.638	-0.07*	
RAS-J total	80.2	13.4	81.9	14.2	0.58	92	.566	0.12	-0.29 to 0.53
JSQLS Psychosocial	45.9	19.1	41.9	19.2	-0.99	92	.324	-0.21	-0.62 to 0.20
JSQLS Motivation/energy	49.5	14.1	46.5	13.7	-1.02	92	.313	-0.21	-0.62 to 0.20
JSQLS Symptoms/side effects	36.1	19.2	31.9	19.1	-1.03	92	.305	-0.22	-0.63 to 0.20

Notes: Brief PANSS = Brief Positive and Negative Syndrome Scale; DTD = Draw to Decision; TMT-A, TMT-B = Trail Making Test, Part A and B; SLOF-J = The Specific Levels of Functioning Scale Japanese Version; RAS-J = The Japanese version of the 24-item Recovery Assessment Scale; JSQLS = The Japanese Schizophrenia Quality of Life Scale; ES = Effect size (continuous variable = Hedges' g, *qualitative variable = φ).

**Table 3:** Intergroup comparison between JTC and non-JTC groups after propensity score matching.

	non-JTC (*n* = 27)		JTC (*n* = 27)		*χ*^2^ value/ *t*-value	*df*	*p*-value	ES	ES 95%CI
	Mean/%, SD		Mean/%, SD						
Age (years)	43.6	10.9	44.4	9.0	0.29	52	.776	0.08	-0.45 to 0.60
Gender (m(%)f)	12 (44.4) 15		13 (48.1) 14		0.07	1	1.00	0.04*	
Years of education	12.3	1.6	12.5	1.5	0.18	52	.546	0.16	-0.37 to 0.69
Duration of illness (years)	11.6	6.7	11.9	6.2	0.19	52	.850	0.05	-0.48 to 0.59
Antipsychotic medication dose (mg)	430.3	286.5	460.2	264.2	0.40	52	.691	0.11	-0.42 to 0.63
Brief PANSS total	15.3	6.9	15.2	6.6	-0.06	52	.952	-0.02	-0.54 to 0.51
Brief PANSS positive	4.1	2.8	4.4	2.8	0.39	52	.696	0.11	-0.42 to 0.63
Brief PANSS negative	6.3	2.6	5.5	2.2	-1.17	52	.245	-0.31	-0.84 to 0.22
Brief PANSS general	4.9	2.5	5.3	2.5	0.54	52	.592	0.14	-0.38 to 0.67
DTD (85:15)	6.6	6.2	1.7	1.0	-4.11	52	.000	-1.10	-1.67 to -0.53
DTD (60:40)	8.3	6.4	1.4	0.5	-5.58	52	.000	-1.50	-2.09 to -0.89
TMT-A	51.1	21.0	49.9	16.1	-0.24	52	.815	-0.06	-0.60 to 0.47
TMT-B	87.7	39.2	86.7	32.0	0.27	52	.785	0.07	-0.45 to 0.60
SLOF-J total	101.0	11.5	93.9	15.0	-1.94	52	.056	-0.52	-1.06 to 0.01
SLOF-J Interpersonal Functioning	26.9	5.0	25.4	5.6	-1.01	52	.319	-0.27	-0.80 to 0.26
SLOF -J Everyday Activities	50.1	4.6	48.9	4.4	-1.00	52	.323	-0.27	-0.79 to 0.26
SLOF-J Vocational Functioning	24.0	4.6	23.4	5.1	-0.42	52	.675	-0.11	-0.64 to 0.41
Housing situation, %	18 (66.7)		17 (63.0)		0.08	1	1.00	-0.04*	
Protected Work, %*	6 (22.2)		7 (25.9)		0.10	1	1.00	0.04*	
Employed, %*	8 (29.6)		9 (33.3)		0.09	1	1.00	0.04*	
RAS total	80.7	11.9	86.3	14.9	1.51	1	.137	0.40	-0.13 to 0.93
JSQLS Psychosocial	45.5	21.2	40.4	22.3	-0.85	51	.396	-0.23	-0.76 to 0.30
JSQLS Motivation/energy	47.6	12.8	45.5	15.7	-0.55	51	.587	-0.15	-0.68 to 0.38
JSQLS Symptoms/side effects	35.4	20.5	26.4	18.5	-1.67	51	.100	-0.45	-0.99 to 0.09

Notes: Brief PANSS = Brief Positive and Negative Syndrome Scale; DTD = Draw to Decision; TMT-A, TMT-B = Trail Making Test, Part A and B; SLOF = The Specific Levels of Functioning Scale Japanese Version; RAS-J = The Japanese version of the 24-item Recovery Assessment Scale; JSQLS = The Japanese Schizophrenia Quality of Life Scale; ES = Effect size (continuous variable = Hedges' g, *qualitative variable = φ).

## 4 Discussion

This study investigated functional outcomes, subjective QoL, and recovery in patients with and without JTC. Participants were in the chronic phase of their disease and exhibited mild psychiatric symptoms. Of the participants, 57.4% were judged to exhibit JTC. Although this was a slightly high percentage, it is within the range reported previously (Dudley et al., 2016).

In step 1, bivariate analyses were conducted to investigate differences between participants with/without JTC. Although there were no significant differences in psychiatric symptoms, those in the JTC group exhibited significantly lower neurocognitive and functional outcomes, with moderate ES. In contrast, there were no significant differences in subjective measures, such as QoL, with small ESs. However, participants in the JTC group were generally older, had fewer years of education, and longer duration of illness.

In step 2, a covariate adjustment via propensity score matching was conducted. Here, the only significant result was for DTD. The ES of functional results was moderate, and ESs for recovery and some symptom-related QoL were small to moderate. Importantly, functional outcomes tended to be lower in the JTC group (moderate ES), while symptom-related QoL and subjective recovery tended to be higher (small to moderate ES). In contrast, ES for neurocognitive function was exceedingly small.

### 4.1 Relationship between JTC and functional outcomes

Regarding the relationship between JTC and functional outcome, this study hypothesised lower functional outcome in the JTC group, similar to Andreou et al. (2014). This study's results (step 1) showed that the JTC group had significantly lower functional outcomes than the non-JTC group, supporting the hypothesis. In contrast, there was no difference in functional achievements (e.g. rates of employment referred to as milestones) between people with and without JTC. This may be because the SLOF assesses functional impairments that do not appear in the milestones (Harvey et al., 2012). Functional outcomes were significantly lower in the JTC group, and ES was moderate. This trend was also true for the sub-items of interpersonal functioning, everyday activities, and vocational functioning. These results suggest that the relationship between JTC and social functioning is not only related to occupational functioning, but also to interpersonal functioning and the domain of daily activities (Andreou et al., 2014). Step 2 also showed a moderate ES for global functioning, although no statistical significance was observed. While samples with similar propensity scores tend to have similar variable distributions (Austin, 2011), this was contradicted by the results of this study, suggesting a consistently low functional outcome for the JTC group.

### 4.2 JTC and recovery

It was hypothesised that subjective measures such as recovery would be lower among participants in the JTC group. However, although not statistically significant, in contrast to expectations, subjective recovery tended to be higher in the JTC group (small to moderate ES). Basically, recovery is positively correlated with functional outcomes (Van Eck et al., 2018). Nevertheless, the results showed that the JTC group trended toward lower functional outcomes and higher recovery. Considering JTC, there may be a negative relationship between functional outcomes and recovery. Such conflicting relationships may be related to subjective overestimation (Sanchez and Dunning, 2020). For instance, the person with schizophrenia is prone to over- or underestimation (Silberstein et al., 2018; Silberstein and Harvey, 2019). Particularly, the tendency to overestimate has been associated with low functional outcome and low social cognition, including JTC (Perez et al., 2020; Silberstein et al., 2018). Given the study's results, the JTC group tends to overestimate and have lower functional outcome (Perez et al., 2020; Silberstein et al., 2018), and may be a good group in terms of subjective indicators. Although no concrete conclusions can be drawn in this regard, this study's results suggest that JTC may have an independent and subjective effect on schizophrenia.

### 4.3 JTC and neurocognitive function

This study hypothesised that the JTC group would have more severe neurocognitive dysfunction than the non-JTC group (Krezolek et al., 2019; Takeda et al., 2018). As hypothesised, before adjustment for covariates, the group with JTC had significantly lower TMT performance. After adjusting for covariates, no intergroup differences were found in neurocognitive function. There were also comparable degrees of impairment in both groups. While the accuracy of the results must be determined with caution, this may be at least partially due to the large age-related and other effects on TMT-J scores (Tombaugh, 2004). Previous studies have repeatedly revealed working memory impairments in participants with JTC; however, sociodemographic factors such as age may have been confounding (Krezolek et al., 2019; Takeda et al., 2018). The results therefore support the opinion that working memory impairments do not constitute definitive explanatory factors for JTC (Evans et al., 2015). However, although TMT is moderately correlated with general neurocognitive assessment (Mazhari et al., 2014), it is not a comprehensive assessment of cognitive function and has limited results.

### 4.4 Limitations

This study has some limitations. First, it is difficult to generalise the results due to the study's cross-sectional design, the recruitment of participants through non-probability sampling, and the possibility of self-selection bias. There is also a lack of evidence for results in groups with more severe psychiatric symptoms. Second, although the propensity scores reduced the observed variable bias, this study was unable to examine the residual confounders caused by biases resulting from differences in unmeasured variables between groups. Specifically, depressive symptoms, which are strongly associated with recovery or self-assessment, were not included (Jones et al., 2020; Van Eck et al., 2018). Third, the sample size was small, with a sample population of 94 people. Therefore, further reduction in the number of cases due to propensity score matching would have reduced power, such that results would have likely been nonsignificant after Bonferroni correction. Therefore, the study should be replicated with a larger sample. Furthermore, due to the large range between the upper and lower limits of the 95% confidence interval for the ES, the ES estimates cannot be considered definitive. Future research must clarify the causal relationship between JTC and functional outcomes/recovery, and the extent of any longitudinal changes in this relationship.

### 4.5 Clinical implications and conclusions

This study is novel in its exploration of JTC and social functions, including the areas of interpersonal, daily life, and employment functioning, and subjective recovery. The results may contribute to social cognitive training, especially training aimed at JTC. First, the JTC group tended to have poor functional outcomes, suggesting that improvements may be achieved through a combination of support types, including cognitive remediation therapy, social skills training, and metacognitive training (Kurtz and Mueser, 2008; Liu et al., 2018; Wykes et al., 2011). Second, although functional outcomes were relatively poor among participants in the JTC group, their subjective ratings tended to be favourable. This suggests that such individuals may not consider their own QoL and recovery to be problematic. Alternatively, they may express that they are ‘not troubled’ or ‘satisfied’, even when experiencing difficulties in social life. Hence, persons who support patients (e.g. social workers and occupational therapists) should consider such aspects on an individual basis through subjective evaluations. Third, there may be a trade-off between poor functional outcomes and good subjective recovery. Thus, programmes designed to improve social cognition and functional outcomes for individuals with JTC may result in poorer recovery. While the cross-sectional nature of this study prevented the direct demonstration of this trade-off relationship, subjective assessments (e.g. recovery levels) may be important indicators in the context of rehabilitation in JTC. Changes in functional outcomes and subjective recovery following rehabilitation for JTC needs to be investigated in longitudinal studies.

## CRediT authorship contribution statement

Seiichi Watanabe: Conceptualisation, methodology, validation, formal analysis, investigation, data curation, writing, original draft preparation, visualisation, and project administration.

Takamichi Taniguchi: Conceptualisation, methodology, resources, and supervision.

Motoko Sugihara: Conceptualisation, methodology, resources, supervision, and project administration.

## Funding

This research did not receive any grants from funding agencies in the public, commercial, or not-for-profit sectors.

## Declaration of competing interest

The authors declare no conflicts of interest associated with this study.
